# COVID-19 Infection and Incidence of Myocarditis: A Multi-Site Population-Based Propensity Score-Matched Analysis

**DOI:** 10.7759/cureus.21879

**Published:** 2022-02-03

**Authors:** Shivani Priyadarshni, Jordan Westra, Yong-Fang Kuo, Jacques G Baillargeon, Wissam Khalife, Mukaila Raji

**Affiliations:** 1 Internal Medicine, University of Texas Medical Branch, Galveston, USA; 2 Epidemiology and Public Health, University of Texas Medical Branch, Galveston, USA; 3 Preventive Medicine, University of Texas Medical Branch, Galveston, USA; 4 Internal Medicine - Geriatrics, University of Texas Medical Branch, Galveston, USA

**Keywords:** covid-19 incidence, cardiac magnetic resonance (cmr), odd ratio, myocarditis, severe acute respiratory syndrome coronavirus 2 (sars-cov-2)

## Abstract

Background

Cardiovascular complications from COVID-19 include myocarditis, acute myocardial infarction, heart failure, and others. Population-level data is lacking about the relationship between COVID-19 and cardiovascular complications; therefore, we conducted a study to examine the incidence of myocarditis, acute myocardial infarction (AMI), heart failure (HF) after COVID-19 infection.

Methods

Retrospective cohort study using de-identified data from 50 health systems across the United States. Cohort groups were created using patients ≥18 who were admitted to hospitals for respiratory illness with COVID-19 in 2020 and respiratory illness without COVID-19 for 2020 and 2019. There were 107,699 patients with COVID-19, 77,499 patients with respiratory illness in 2020, and 112,898 patients in 2019. The COVID-19 group was matched to each respiratory illness group by propensity score. Patients with prior specific cardiovascular events such as myocarditis, AMI, HF were excluded. The primary outcome was myocarditis, and secondary outcomes were AMI and HF.

Results

In the COVID-19 group, 79 (0.12%) patients had new-onset myocarditis compared to 29 (0.04%) patients in the non-COVID-19 control (Pneumonia/flu) group Odd's Ratio (OR), (OR 2.73, CI 95%, 1.78-4.18). In the COVID-19 group, 1512 patients developed HF compared to 2,659 patients in the non-COVID-19 group (OR 0.49, CI 95%, 0.46-0.52). 1125 patients in COVID-19 group had AMI compared to 1243 patients in non-COVID-19 group (OR 0.87, CI 95%, 0.80-0.94).

Conclusion

COVID-19 was associated with a 2-3-fold higher risk of myocarditis. Unexpectedly, lower rates of HF diagnosis reflect challenges faced due to the severity of lung disease leading to obscuring physical exam findings required for HF diagnosis and early mortality before a diagnosis of HF was made.

## Introduction

COVID-19 affects millions of people worldwide and has been declared a pandemic by The World Health Organization in 2020 [1,2]. COVID-19-infection has protean manifestations ranging from asymptomatic infection to multi-organ failure and death. COVID-19 primarily affects the respiratory system; however, increasing multi-organ complications have been identified over time [3]. In particular, a wide range of cardiovascular complications has been linked to COVID-19, including myocarditis, acute myocardial infarction (AMI), heart failure (HF), life-threatening arrhythmias, cardiogenic shock, etc. Cardiovascular manifestations were found in 20-30% of hospitalized COVID-19 patients and were associated with adverse outcomes [4,5]. Some studies suggest that, among COVID-19 associated cardiovascular complications, myocardial injury is relatively common-accounting for 7%-23% of the cases and is associated with a higher rate of morbidity and mortality [1]. In another study, the cardiac injury ranged from 7% to 17% of hospitalized patients [6,7]. The mechanism of cardiovascular complication is unclear as it is attributed to a combination of direct viral injury to myo-pericardium and the inflammatory cytokine storm. The direct viral injury is postulated via the virus utilizing angiotensin-converting enzyme-2 (ACE2) receptors within cardiac tissue, leading to a COVID-19 induced inflammatory response and increased cytokine storm. Population-level data lacks the relationship between COVID-19 and incident myocarditis, AMI, and HF. We, therefore, examined these associations in an extensive national database.

## Materials and methods

Data source

This study used data from the TriNetX COVID-19 Research Network, which consists of electronic health records (EHR) from 50 health care organizations across the United States. This EHR research network contains patient demographics, diagnoses, procedures, labs, hospital stays, and medications. The population of interest for this study included all adults aged 18 and older who had records in the system. The analyses of these data were conducted from January through April 2021. The University of Texas Medical Branch Institutional Review Board reviewed and approved this study, approval number: 20-0180. Patients who had a previous history of myocarditis, AMI, and HF were excluded from the study.

Cohort

Three cohorts were created for comparison. The first cohort was defined as patients who had a hospitalization that coincided with a COVID-19 diagnosis, International Classification of Disease (ICD), (ICD-9-CM: U07.1) or a positive COVID-19 lab test (Logical Observation Identifiers Names and Codes [LOINC]: 94500-6, 94315-9, 94309-2, 94533-7, 94534-5, 94559-2) in 2020. The second cohort was defined as COVID- 19 negative patients hospitalized for pneumonia (ICD-10-CM: J12.x-J18.x) or influenza (ICD-10-CM=J09.x-J11.x) in 2020, and the third cohort was defined as COVID-19 negative patients who had a hospitalization for pneumonia or influenza in 2019. All patients were required to have at least one visit present in the EHR in the year before hospitalization to be included.

Variables

The primary outcome of interest was the incidence of myocarditis in the three months following hospitalization. Myocarditis was identified by ICD-10-CM codes I40.x, I41.x, or I51.4. Secondary outcomes assessed in the three-month follow-up period were heart failure (ICD-10-CM=I50.x) and acute myocardial infarction (ICD-10-CM= I21.x). The primary independent variable was the inclusion in the COVID-19 cohort versus each of pneumonia/influenza cohorts. Other variables of interest included demographic factors, prior diagnoses, prior procedures, and prior medications. Demographic variables of interest were age, sex, race, and ethnicity. Diagnosis of interest were ischemic heart disease (ICD-10: I20.and-I25.x), pulmonary heart disease (ICD-10: I 26.x-I28.x), other heart disease (ICD-10: I30.x-I52.x), essential hypertension (ICD-10: I10.x), diabetes (ICD-10: E08.x-E13.x), and overweight/obesity (ICD-10: E66.x) in the 12 months before COVID-19, pneumonia, or influenza hospitalization. Procedures of interest were chest x-ray, electrocardiogram, 2-D echocardiogram (2-D ECHO), therapeutic cardiovascular services and procedures, and bypass procedures. Medications of interest were adrenal corticosteroids, beta-blockers, aspirin, heparin, enoxaparin, angiotensin-converting enzyme (ACE) inhibitors, angiotensin II inhibitors, clopidogrel, and spironolactone. Both procedures and medications also occurred 12 months before the index hospitalization.

Statistical analysis

Two comparisons were made for each outcome. The concurrent comparison groups were patients hospitalized for COVID-19 vs. those hospitalized for non-COVID-19 pneumonia/influenza (COVID-19 negative controls) in 2020. We also compared patients hospitalized for COVID-19 vs. those hospitalized for pneumonia/influenza in 2019. Our focus was on comparing 2020 COVID-19 to 2020 pneumonia/influenza. Still, we also examined the 2019 pneumonia/influenza to see if there is any substantial year-to-year change in the prevalence of pneumonia/influenza. Propensity score matching was done to balance the cohorts using the demographic, diagnosis, procedure, and medication variables listed above for each comparison group. For examining the incidence of each outcome, patients who had the outcome in the year before their hospitalization were removed from analysis of that outcome. Using the propensity-matched cohorts, logistic regression was used to obtain odds ratios (OR) and 95% confidence intervals (CI) for each of the three outcomes. These odds ratios compared the odds of each of the outcomes (myocarditis, heart failure, or AMI) between the COVID-19 group and each of the pneumonia/influenza groups (2019 or 2020). We also compared the proportion of patients who received troponin tests (vs. those who did not) and the difference in troponin levels among those who received the tests between the two groups for each propensity score-matched cohort, using logistic regression and t-test, respectively. All statistical analyses were conducted with the TriNetX platform, which utilizes a combination of JAVA, R, and Python programming languages.

## Results

There were a total number (N) of 107,699 patients in the COVID-19 group and 77,499 patients in the 2020 pneumonia/influenza group. The mean age of the patient population in the COVID-19 group was 59.99 [Standard Deviation (SD) 17.86] and 64.70 (SD 17.19) in the pneumonia/influenza group in 2020. There was an almost equal distribution of gender among these two groups. Among the COVID-19 group, 58.07% were white patients, and in the pneumonia/influenza 2020 group, the white patient population was 68.85%. Most patients among these groups were non-Hispanics. Among diagnoses, ischemic heart disease was present in 14.16% of patients in the COVID-19 group and 25.20% in pneumonia/influenza group 2020. Pulmonary heart disease was present in 4.49% of patients in the COVID-19 group and 9.81% of patients in pneumonia/influenza group in 2020. Essential Hypertension (HTN) was the most prevalent diagnosis among the groups; it was found in 34.40% of COVID-19 patients and 45.44% of the pneumonia/influenza group 2020. Diabetes Mellitus (DM) was found in 22.39% in the COVID-19 group and 26.68% in the pneumonia/influenza group 2020. Obesity and overweight had similar distributions among these two groups. Chest radiological exams were performed in 24.12% and 38.87% of patients in the Pneumonia/influenza 2020 group. There was a total of 11,2989 patients in the Pneumonia/Influenza 2019 group and 77,499 patients in the 2020 pneumonia/influenza group. In the 2020 pneumonia/influenza group, fewer patients had pneumonia/influenza, which could be attributed to regular and mandatory use of personal protective equipment such as masks during the pandemic. Compared to the 2019 cohort, the average age was about half a year younger for the 2020 cohort. Table 1 represents the baseline characteristics of patients before matching.

**Table 1:** Represents the baseline characteristics of patients before propensity matching Number = N; Standard Deviation = SD

		COVID-19		Pneumonia/Influenza 2019		Pneumonia/Influenza 2020	
		N	Percent	N	Percent	N	Percent
ALL		1,07,699	100%	1,12,898	100%	77,499	100%
Demographics							
Age at Index (mean, SD)		59.99	17.86	65.24	17.46	64.7	17.19
Sex	Male	53,873	49.95%	57,311	50.76%	40,651	52.42%
	Female	53,826	49.90%	55,587	49.23%	36,848	47.51%
Race	White	62,631	58.07%	79,550	70.46%	53,392	68.85%
	Black or African American	20,155	18.69%	15,367	13.61%	11,534	14.87%
	Asian	2,241	2.08%	1,464	1.30%	976	1.26%
	American Indian or Alaska Native	1,207	1.12%	552	0.49%	335	0.43%
	Native Hawaiian or Other Pacific Islander	170	0.16%	89	0.08%	65	0.08%
	Unknown Race	21,460	19.90%	15,884	14.07%	11,252	14.51%
Ethnicity	Not Hispanic or Latino	67,282	62.38%	66,525	58.92%	49,944	64.40%
	Hispanic or Latino	17,312	16.05%	6,039	5.35%	3,855	4.97%
	Unknown Ethnicity	23,270	21.57%	40,342	35.73%	23,755	30.63%
Diagnoses							
Ischemic heart diseases		15,269	14.16%	28,642	25.37%	19,543	25.20%
Pulmonary Heart Disease		4,838	4.49%	10,556	9.35%	7,605	9.81%
Other forms of heart disease		25,510	23.65%	48,294	42.77%	33,557	43.27%
Essential (primary) hypertension		37,110	34.40%	52,150	46.19%	35,243	45.44%
Diabetes mellitus		24,154	22.39%	30,464	26.98%	20,689	26.68%
Overweight and obesity		15,314	14.20%	16,094	14.25%	11,222	14.47%
Procedures							
Radiologic examination, chest		26,013	24.12%	42,982	38.07%	30,146	38.87%
Electrocardiogram		26,174	24.27%	42,279	37.45%	28,863	37.22%
Echocardiography		9,806	9.09%	22,293	19.75%	15,334	19.77%
Therapeutic Cardiovascular Services and Procedures		410	0.38%	817	0.72%	631	0.81%
Bypass		151	0.14%	360	0.32%	278	0.36%
Medications							
Adrenal Corticosteroids		35,683	33.08%	47,044	41.67%	33,096	42.68%
Beta-Blockers/Related		24,313	22.54%	37,475	33.19%	26,685	34.41%
Aspirin		18,584	17.23%	30,196	26.74%	20,123	25.95%
Heparin		14,403	13.35%	25,908	22.95%	18,055	23.28%
Enoxaparin		15,201	14.09%	22,298	19.75%	16,864	21.75%
ACE Inhibitors		11,987	11.11%	17,110	15.15%	11,777	15.19%
Angiotensin II Inhibitor		9,400	8.72%	11,222	9.94%	8,003	10.32%
Clopidogrel		4,603	4.27%	7,549	6.69%	5,382	6.94%
Spironolactone		3,362	3.12%	5,806	5.14%	4,196	5.41%

Two comparison groups were created post matching. 1) 2020 Comparison group (Comparison of COVID-19 cohort with Pneumonia/influenza cohort 2020), 2) 2019 Comparison group (Comparison of COVID-19 cohort with Pneumonia/influenza cohort 2019). The difference among characteristics between COVID-19 and pneumonia/influenza cohorts was very minimal post matching. In the 2020 comparison group, the mean age of COVID-19 patients was 64.03 (SD 16.90), and the mean age in Pneumonia/influenza group was 63.64 (SD 17.52). The most prevalent diagnosis among the 2020 comparison group was HTN, 42.33% in the COVID-19 group and 41.53% in the Pneumonia/influenza group, followed by a diagnosis of other forms of heart disease; 34.84% in the COVID-19 group and 34.99% in the Pneumonia/influenza group. A radiological exam of the chest was performed in 32.84% in the COVID-19 group and 32.66% in the Pneumonia/influenza group. An electrocardiogram was done 31.97% in the COVID-19 group and 31.59% in the Pneumonia/influenza group. Corticosteroids were used in 39.34% of patients in the COVID-19 group and in 38.85% of patients in the Pneumonia/influenza group, which can be attributed to corticosteroids mainstay of treatment in COVID-19 infection. Table 2 represents the baseline characteristic of patients' post propensity score matching among different cohorts.

**Table 2:** Represents the baseline characteristic of patients' post propensity score matching among different cohorts. Number = N; Standard Deviation = SD

Patient Characteristics - After Matching		2019 Comparison				2020 Comparison			
		COVID		Pneumonia/Influenza		COVID		Pneumonia/Influenza	
		N	Percent	N	Percent	N	Percent	N	Percent
		75,067	100%	75,065	100%	63,652	100%	63,653	100%
Demographics									
Age at Index (mean, SD)		63.14	17.14	62.8	17.95	64.03	16.9	63.64	17.52
Sex	Male	38,103	50.76%	38,339	51.07%	33,250	52.19%	32,940	51.71%
	Female	36,964	49.24%	36,726	48.92%	30,402	47.72%	30,713	48.21%
Race	White	47,432	63.18%	48,156	64.15%	41,126	64.56%	41,406	64.99%
	Black or African American	12,131	16.16%	12,149	16.18%	10,683	16.77%	10,210	16.03%
	Asian	1,212	1.61%	1,191	1.59%	882	1.38%	933	1.47%
	American Indian or Alaska Native	545	0.73%	511	0.68%	359	0.56%	325	0.51%
	Native Hawaiian or Other Pacific Islander	90	0.12%	77	0.10%	65	0.10%	62	0.10%
	Unknown Race	13,663	18.20%	12,989	17.30%	10,592	16.63%	10,771	16.91%
Ethnicity	Not Hispanic or Latino	48,342	64.39%	49,124	65.44%	42,804	67.19%	41,922	65.80%
	Hispanic or Latino	5,708	7.60%	5,820	7.75%	3,390	5.32%	3,835	6.02%
	Unknown Ethnicity	21,023	28.00%	20,129	26.81%	17,513	27.49%	17,950	28.18%
Diagnoses									
Ischemic heart diseases		13,720	18.28%	13,945	18.58%	12,906	20.26%	12,998	20.40%
Pulmonary Heart Disease		4,440	5.91%	4,647	6.19%	4,308	6.76%	4,494	7.05%
Other forms of heart disease		23,161	30.85%	23,547	31.37%	22,193	34.84%	22,290	34.99%
Essential (primary) hypertension		29,961	39.91%	29,895	39.82%	26,970	42.33%	26,457	41.53%
Diabetes mellitus		18,419	24.54%	18,607	24.79%	16,421	25.78%	16,102	25.28%
Overweight and obesity		10,511	14.00%	10,438	13.90%	9,178	14.41%	9,060	14.22%
Procedures									
Radiologic examination, chest		22,519	30.00%	23,051	30.71%	20,924	32.84%	20,806	32.66%
Electrocardiogram		22,350	29.77%	22,745	30.30%	20,365	31.97%	20,123	31.59%
Echocardiography		9,274	12.35%	9,724	12.95%	8,921	14.00%	9,236	14.50%
Therapeutic Cardiovascular Services and Procedures		385	0.51%	404	0.54%	375	0.59%	381	0.60%
Bypass		135	0.18%	133	0.18%	132	0.21%	143	0.22%
Medications									
Adrenal Corticosteroids		27,946	37.23%	28,257	37.64%	25,059	39.34%	24,752	38.85%
Beta Blockers/Related		20,326	27.08%	20,716	27.59%	19,093	29.97%	18,871	29.62%
Aspirin		15,783	21.02%	16,036	21.36%	14,327	22.49%	14,182	22.26%
Heparin		12,679	16.89%	12,863	17.13%	11,930	18.73%	11,985	18.81%
Enoxaparin		12,450	16.58%	12,519	16.68%	11,918	18.71%	11,794	18.51%
Ace Inhibitors		9,676	12.89%	9,788	13.04%	8,731	13.71%	8,681	13.63%
Angiotensin II Inhibitor		7,054	9.40%	7,093	9.45%	6,391	10.03%	6,257	9.82%
Clopidogrel		3,985	5.31%	4,040	5.38%	3,713	5.83%	3,723	5.84%
Spironolactone		2,887	3.85%	2,983	3.97%	2,758	4.33%	2,785	4.37%

Comparison between COVID-19 and Pneumonia/influenza 2020 groups: In the COVID-19 group, 79 patients had new-onset myocarditis compared to 29 patients in the non-COVID-19 control (Pneumonia/influenza 2020) group (OR 2.73, CI 95%, 1.78-4.18). In the COVID-19 group, 1512 patients developed HF compared to 2,659 patients in the non-COVID-19 group (OR 0.49, CI 95%, 0.46-0.52). 1125 patients in COVID-19 group had AMI compared to 1243 patients in non-COVID-19 group (OR 0.87, CI 95%, 0.80-0.94). These results were robust in comparing COVID-19 and Pneumonia/influenza 2019. In addition, we found that COVID-19 patients received more troponin tests but had a lower level of troponin than pneumonia/influenza patients in 2020. Table 3 represents an analysis of cardiac outcomes for the COVID-19 group to Pneumonia/influenza 2019 and 2020 groups, respectively.

**Table 3:** Represents an analysis of cardiac outcomes for the COVID-19 group to Pneumonia/influenza 2019 and 2020 groups, respectively Number = N; Standard Deviation = SD; Odds Ratio = OR; Confidence Interval = CI; Acute Myocardial Infarction = AMI REF = Reference for the comparison. For each of the odds ratios, we are comparing the COVID 19 group to the pneumonia/flu group, which is considered the reference group.

Outcomes	2019 Comparison				2020 Comparison			
	Myocarditis				Myocarditis			
	N	With Outcome	OR (95% CI)		N	With Outcome	OR (95% CI)	
Pneumonia /Influenza	74,888	39	REF		63,515	29	REF	
COVID-19	74,807	90	2.31 (1.59, 3.37)		63,451	79	2.73 (1.78, 4.18)	
	Heart Failure				Heart Failure			
	N	With Outcome	OR (95% CI)		N	With Outcome	OR (95% CI)	
Pneumonia /Influenza	52,250	3,329	REF		43,001	2,659	REF	
COVID-19	58,817	1,745	0.45 (0.42, 0.48)		48,569	1,512	0.49 (0.46, 0.52)	
	AMI				AMI			
	N	With Outcome	OR (95% CI)		N	With Outcome	OR (95% CI)	
Pneumonia /Influenza	65,988	1,322	REF		54,790	1,243	REF	
COVID-19	67,691	1,300	0.96 (0.89, 1.03)		56,870	1,125	0.87 (0.80, 0.94)	
	Troponin Test				Troponin Test			
	N	Had Troponin Test	OR (95% CI)		N	Had Troponin Test	OR (95% CI)	
Pneumonia /Influenza	75,073	10,389	REF		63,707	7,341	REF	
COVID-19	75,073	8,572	0.80 (0.78, 0.83)		63,707	7,673	1.05 (1.02, 1.09)	
	Troponin Values				Troponin Values			
	N	Mean (SD)		p-value	N	Mean (SD)		p-value
Pneumonia /Influenza	8,158	3.28 (29.20)		< .0001	6,261	2.07 (25.63)		0.0022
COVID-19	7,427	1.00 (9.76)		< .0001	6,627	1.02 (10.50)		0.0022

## Discussion

Our study was a population-based propensity score-matched analysis that used real-world COVID-19 data from EHR from 50 health care organizations in the United States. We identified two propensity score-matched cohorts, persons diagnosed: COVID-19, non-COVID-19 cohort 2020 (Pneumonia/Influenza), and COVID-19 to non-COVID-19 cohort 2019 (Pneumonia/Influenza). Our study showed an incidence of myocarditis among COVID-19 patients that were 2 to 3 times higher than non-COVID-19 controls.

Previous studies have shown a higher and varying range of incidence of myocarditis: a study of 150 patients in Wuhan by Ruan et al. reported 7% of deaths were due to myocardial damage-causing circulatory failure, with 33% of the patients dying of both myocardial damage and circulatory failure [8]. Another study by Siripanthong et al. showed 7% of COVID-19-related deaths were attributable to myocarditis [9]. Other studies showed myocardial injury is relatively common-accounting for 7%-23% of cases [10].

The incidence of myocarditis in our study was 12.5 per 10,000. Our rate was lower than previously reported rates of 2.3-33% in previous studies, which are mostly done on patients in the early phase of the pandemic. Our explanation for the difference could be related to our larger sample size and increasing availability of interventions (e.g., steroids, antibodies, Remdesivir) at the later phases of the COVID, especially in the USA. An increase in the incidence (2.3%) of myocarditis was found in a cohort study of 1597 US competitive athletes with Cardiovascular Magnetic Resonance Imaging (CMR) screening after COVID-19 infection by Daniels et al. [11]. Unlike the study done on athletes, our study population represented real-world patients with multiple chronic conditions (e.g., diabetes, obesity) known to increase the risk and severity of COVID-19. Our study findings are consistent with previous studies; however, our incidence rate was lower, which could be attributed to various factors: larger population size, using ICD codes to diagnose myocarditis, lack of use of cardiovascular magnetic resonance imaging to diagnose myocarditis, among others. Also, the incidence reported in previous studies is assumed and not based on confirmatory diagnoses such as autopsies or use of CMR-which thus might represent overestimation. Certain ethnic groups can be disproportionately affected by SARS-CoV-2, and studies done outside of the United States of America (USA) may not represent the ethnic and racial diversity in the USA. For example, COVID-19 morbidity and death rates were higher among African Americans than other ethnicities in many American states. So, the difference in our myocarditis incidence rates compared to other studies could also reflect different populations; for example, our patients' mean age post matching was 63+/- 2 compared to the average age of 22 in the previously mentioned paper in the Daniels Elite Athlete study.

Unexpected findings in our study are the lower incidence of HF and the lower troponin level values in hospitalized COVID-19 patients compared with hospitalized PS-matched non-COVID-19 Pneumonia/influenza patients. The reasons for these findings are unclear. This may suggest that troponins are not fully capturing myocardial injury in COVID-19 patients, underscoring a need for more use of CMR to diagnose early myocardial injury/myocarditis (followed by timely interventions) and therefore reducing late sequelae of a COVID-19-stunned myocardium. It is also possible that the lower rate of new HF diagnoses may reflect challenges faced due to the severity of lung disease among these patients. For example, HF was diagnosed less as the respiratory disease due to COVID was so severe that physical exam findings such as crackles or other exam findings were obscure in the setting of severe lung disease. Another explanation includes the death of severely sick patients due to respiratory causes before a diagnosis of heart failure was made. Also, a lower rate of use of 2-D ECHO and other high-touch diagnostics in hospitalized COVID-19 patients kept in strict isolation with hospital staff in full protective gear- a needed measure to mitigate COVID-19 spread in the hospital. There might be an artificially lower rate of HF diagnoses in the COVID-19 group in this possible scenario. While data exist on the psychological effects of COVID-19-related isolation, very little is known on how the isolation affects diagnostics (e.g., 2-D ECHO and CMR) and interventions (e.g., physical therapy) for isolated COVID-19 patients [12]. We, however, do not have the data to examine this possible scenario. Still, it is an important area for future research-vis-à-vis how to optimize needed testing and interventions in the context of patient isolation and thus improve early detection and timely treatment of potentially treatable cardiac and other complications in hospitalized patients. Similar findings of less testing, less care, and more adverse outcomes in patients isolated for other reasons have been reported [13,14]. Abad et al. showed that physicians and nurses spent less time with isolated patients, a practice linked to adverse patient safety outcomes, including an eight-fold increase in adverse care events due to suboptimal supportive care measures [13]. We do not explain the increase in troponin values in the non-COVID-19 group. However, demand ischemia, hypoxia, and other sources of troponins such as pulmonary embolism, etc., might have contributed to the elevation in troponins. This is another area for future study.

The mechanisms by which COVID-19 causes cardiovascular damage are multifactorial; protean cardiovascular manifestations of COVID-19 infections reported in the literature range from myocarditis, heart failure, AMI, cardiac arrhythmias, and cardiogenic shock. It is not surprising that myocarditis is a sequel of COVID-19, given that myocardial cells are a potential target of SARS-CoV-2. Cardiac injury, defined as elevated troponins, relates to leukocytosis, elevated serum ferritin level, and inflammatory markers such as interleukin-6 (IL-6) and C reactive protein (CRP), suggesting an important correlation between myocardial injury and inflammatory hyperactivity caused by viral infection [15]. It is known that other non-COVID-19 viral infections such as the influenza virus and others can cause damage to cardiac tissue [16]. However, the incidence of myocardial injury is suspected to be higher with COVID-19 infection than with other viral infections-most likely attributed to direct viral damage via ACE 2 receptors within the cardiac tissue.

Initial workup for myocarditis involves checking biomarkers such as troponins and obtaining an electrocardiogram (EKG). EKGs are usually abnormal in patients with myocarditis, but EKG findings are not specific. Similarly, troponin elevation is seen in myocarditis, but troponins could also be elevated due to several other causes, including demand ischemia. An echocardiogram is the essential first-line noninvasive test in the workup for myocarditis. Common transthoracic echocardiogram findings include global left ventricular hypokinesis, regional wall motion abnormalities, and dilated or hypertrophic ventricles [17]. Cardiac magnetic resonance (CMR) imaging is important in diagnosing myocarditis, especially if an endomyocardial biopsy is unavailable or obtained [18]. Endomyocardial biopsy is the gold standard test to diagnose myocarditis; however, it is invasive and seldomly performed if there is an ongoing COVID-19 infection and the patient is isolated. The mainstream treatment of COVID-19 induced myocarditis is supportive therapy. There is no clear data or guidelines available for the use of steroids monotherapy and immunomodulators such as Interleukin (IL)-6 inhibitors monotherapy or combination therapy. However, some case reports have suggested some benefits from using steroids and immunomodulators such as IL-6 inhibitors to treat COVID-19 myocarditis [19]. It is essential to understand that myocarditis is a known sequela of COVID-19 infection and is linked to a substantial increase in morbidity, post-COVID-19 disability, and mortality.

Therefore, early recognition of myocarditis (and other cardiac sequelae of COVID-19) and prompt formation of a multidisciplinary intervention team is required to prevent rapid deterioration and adverse outcomes in these patients.

Limitations

There were several limitations to our study: Although propensity score matching made the demographic and disease characteristics between the COVID-19 and pneumonia/influenza groups more comparable, our analyses lost about 30-40% of COVID-19 patients due to match. The post matching COVID-19 cohort was older and had a higher proportion of Whites and comorbidities than the COVID-19 cohort pre-match, which might impact the generalizability of study results; The diseases, medical conditions, procedures, and other variables examined in this study were based on ICD-10-CM codes, which are not always accurate or complete. Also, signs and symptoms are not coded with ICD-10-CM codes, so our study did not have data related to the signs and symptoms of the outcomes; The TriNetX database does not represent the general U.S population but rather represents people who receive medical care within the 50 health care organizations in the network; Our study relies on de-identified EHR data, which does not contain data on region and indicators of wealth, poverty, incomes, and other socioeconomic disadvantages, good care for pre-existing cardiovascular disease and other conditions (e.g., diabetes), and access to timely testing and care for COVID-19 and influenza/ pneumonia. 

Despite these limitations, this study had several strengths, including a large sample from 50 health systems covering all major regions of the United States. We also used rigorous analyses based on propensity score matching on both concurrent and past controls to demonstrate the robustness of study results.

## Conclusions

The incidence of myocarditis was 2 to 3 higher in COVID-19 patients than in non-COVID-19 controls. COVID-19 infection had a higher odds of (2-3 times ) of developing myocarditis than non-COVID-19 infections ( flu/pneumonia). Unexpected findings were the lower rates of HF diagnoses in the COVID-19 group. It is also possible that the lower rate of new HF diagnoses may reflect challenges faced due to the severity of lung disease among these patients. For example, HF was diagnosed less as the respiratory disease due to COVID was so severe that physical exam findings such as crackles, or other exam findings were obscure in the setting of severe lung disease. Another explanation includes the death of severely sick patients due to respiratory causes before a diagnosis of heart failure was made.
